# The Effects of Exercise on Fluorosis: A Comprehensive Multisystem Review

**DOI:** 10.3390/vetsci13050446

**Published:** 2026-05-01

**Authors:** Fengge Han, Xiaohui Li, Sheraz Ahmad, Qi Lei, Zilong Sun

**Affiliations:** 1 Physical Education Department, Xinzhou Normal University, Xinzhou 034000, China; hfg85826@126.com; 2 College of Veterinary Medicine, Shanxi Agricultural University, Jinzhong 030801, China; lixiaohuiyayy@outlook.com (X.L.); sherazahmad4582@gmail.com (S.A.); 20231092@stu.sxau.edu.cn (Q.L.)

**Keywords:** fluorosis, physical exercise, oxidative stress, animal models, multi-organ toxicity, livestock health

## Abstract

Fluorosis, a systemic condition caused by chronic excessive fluoride intake, poses significant threats to livestock health and agricultural productivity worldwide. This systematic review synthesizes current evidence on the modulatory effects of exercise against fluorosis, integrating human studies, animal experiments, and methodological considerations. Human studies indicate negative associations between fluoride exposure and cognitive development, muscle function, and exercise capacity, with exercise influencing fluoride pharmacokinetics in an exercise-intensity-dependent manner. Animal experiments consistently demonstrate that regular moderate-intensity exercise attenuates fluoride-induced damage across multiple organ systems through activation of the Nrf2/ARE antioxidant pathway, modulation of BMP-2/Smads and OPG/RANKL/RANK signaling, suppression of inflammatory responses, and preservation of intestinal barrier integrity. Substantial heterogeneity exists among current fluorosis models regarding exposure dosages, durations, and exercise protocols, underscoring the need for standardization and consideration of genetic background. Overall, exercise shows promise for mitigating fluorosis-induced multi-organ damage, although human evidence remains limited. Future research should prioritize model optimization, elucidation of molecular targets, and exploration of synergistic interventions to provide a foundation for veterinary clinical management.

## Simple Summary

Fluorosis, caused by chronic excessive fluoride exposure, poses a significant health threat to livestock worldwide, characterized by skeletal deformities, lameness, reduced growth, and decreased productivity. This review examines whether exercise can mitigate fluoride toxicity. Evidence from animal studies demonstrates that regular moderate-intensity exercise protects multiple organ systems, including the brain, liver, bone, intestine, heart, kidneys, and teeth-by reducing oxidative stress, attenuating inflammation, and inhibiting apoptosis. Exercise also preserves bone health and maintains intestinal barrier function. Human studies remain limited but suggest that exercise modulates fluoride absorption and retention. Notably, fluoride exposure itself impairs muscle function and exercise capacity, creating a complex bidirectional relationship. Understanding exercise–fluorosis interactions may support cost-effective strategies to improve animal health in fluoride-endemic regions.

## 1. Introduction

Fluorosis is a chronic toxicological condition resulting from prolonged excessive fluoride exposure and remains a significant environmental and veterinary health concern worldwide [1,2,3]. Although low concentrations of fluoride are beneficial for dental health, sustained intake above physiological thresholds leads to cumulative tissue deposition and progressive systemic dysfunction. Endemic fluorosis affects millions of individuals globally, particularly in regions where groundwater fluoride concentrations exceed recommended limits [4,5,6]. Importantly, fluorosis is not restricted to human populations; numerous cases have been documented in livestock species-including cattle, sheep, goats, and horses-raised in areas with fluoride-contaminated water or forage [7].

Skeletal fluorosis represents the most clinically severe manifestation. Owing to fluoride’s strong affinity for calcium ions, fluoride replaces hydroxyl groups within hydroxyapatite crystals, forming fluorapatite and altering bone crystallinity [8,9]. This substitution increases mineral density but compromises structural integrity, leading to osteosclerosis, joint stiffness, lameness, and reduced mobility [4,5]. In livestock systems, these skeletal abnormalities are associated with decreased grazing efficiency, reduced weight gain, impaired reproduction, and diminished productivity [7]. Thus, fluorosis represents both a toxicological and an economic burden in veterinary medicine. Beyond skeletal tissues, fluoride exerts multisystem effects. Molecular studies demonstrate that fluoride inhibits key metabolic enzymes through magnesium–fluoride complex formation, disrupts glycolysis and ATP production, induces mitochondrial dysfunction, and generates excessive reactive oxygen species (ROS) [10,11,12,13,14]. Oxidative stress and redox imbalance have emerged as central mechanisms underlying hepatic, renal, neural, and bone pathology in fluorosis [11,15].

Clinical fluoride exposure levels—In humans, chronic fluoride toxicity typically occurs when drinking water contains fluoride above 1.5 mg/L (the WHO guideline value), with skeletal fluorosis developing after 10–20 years of cumulative exposure at concentrations >4 mg/L [4,5]. In livestock, tolerable levels are lower: for cattle, long-term dietary fluoride intake exceeding 30–40 mg/kg dry matter is associated with dental and skeletal lesions, reduced weight gain, and decreased productivity [7]. These thresholds provide a reference for experimental models, although the majority of rodent studies use much higher concentrations (50–100 mg/L NaF in drinking water), which may limit direct extrapolation to field conditions.

Physical exercise induces broad physiological adaptations that may counteract several of these pathological processes. Mechanical loading enhances bone remodeling through activation of Wnt/β-catenin and BMP-Smad signaling pathways [16,17,18], while moderate exercise strengthens antioxidant defenses via Nrf2/ARE-mediated transcriptional regulation [19,20]. Exercise also promotes mitochondrial biogenesis, improves glucose metabolism, and attenuates chronic inflammation [21,22,23,24,25,26,27]. These systemic effects suggest a potential modulatory role for exercise in fluoride toxicity.

The novelty of this review lies in: (i) systematically integrating human and animal evidence for a bidirectional exercise–fluorosis relationship; (ii) proposing a multi-organ protective framework centered on common molecular pathways; and (iii) critically appraising the translational gap between high-dose rodent models and real-world veterinary exposure. Most animal studies use fluoride doses (50–100 mg/L) far exceeding typical environmental levels; thus, findings should be interpreted with caution. Although several rodent studies report that exercise alleviates fluoride-induced oxidative stress, apoptosis, and remodeling imbalance [4,28,29,30,31], a coherent synthesis integrating human data, animal mechanisms, model design, and veterinary relevance has not been comprehensively presented. Furthermore, the reciprocal question—whether fluoride toxicity impairs exercise capacity—remains largely unexplored.

**Figure 1:**
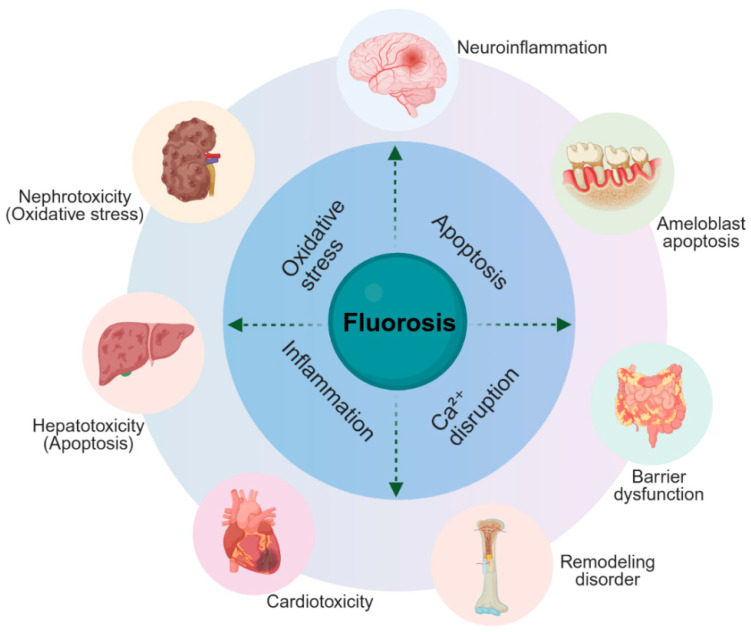
Circular overview of fluoride-induced multi-organ toxicity. Fluoride triggers core mechanisms (oxidative stress, apoptosis, inflammation, Ca^2+^ disruption) leading to damage in seven organs. Key pathological features are indicated. This schematic introduces the organ-specific effects detailed in Section 3.

Accordingly, this review aims to summarize current human evidence on exercise and fluoride pharmacokinetics while analyzing animal studies to elucidate organ-specific mechanisms. Figure 1 schematically illustrates the core mechanisms and multi-organ manifestations of fluoride toxicity, providing a foundation for understanding the protective effects of exercise detailed in Section 3. It also evaluates the experimental models employed to investigate exercise–fluoride interactions and discusses the veterinary implications, including the potential effects of fluorosis on physical performance, as well as future research directions.

## 2. Search Strategy and Study Selection

This review was conducted following the PRISMA (Preferred Reporting Items for Systematic Reviews and Meta-Analyses) guidelines. A systematic literature search was performed in PubMed, Web of Science, and Scopus databases for articles published up to January 2026. The search strategy used combinations of the following keywords: (“fluorosis” OR “fluoride toxicity”) AND (“exercise” OR “physical activity”) AND (“animal model” OR “rodent” OR “human”) AND (“oxidative stress” OR “apoptosis” OR “inflammation” OR “bone remodeling”). Additional relevant studies were identified by manually screening reference lists of included articles.

Inclusion criteria were: (i) original research or systematic reviews investigating the effects of exercise on fluoride toxicity in animals or humans; (ii) studies reporting quantitative outcomes related to organ damage, oxidative stress, inflammation, apoptosis, or fluoride metabolism; (iii) English language articles. Exclusion criteria were: conference abstracts, case reports, commentary articles, and studies without proper control groups.

A total of 84 relevant studies were ultimately included in this review. No meta-analysis was performed due to substantial heterogeneity in exposure protocols, outcome measures, and experimental designs.

## 3. Human Studies on Exercise and Fluorosis

Human studies are essential for understanding the health effects of fluoride; however, due to variations in exposure doses, individual differences, confounding factors, and ethical constraints, the relevant evidence is primarily derived from cross-sectional surveys, prospective cohort studies, and a limited number of intervention trials. Existing research encompasses multiple domains, including neuropsychiatric health, sports performance, muscle function, bone metabolism, and the respiratory system, with preliminary explorations into the effects of exercise on fluoride pharmacokinetics [32,33]. Overall, the negative association between fluoride exposure and cognitive development in children has been corroborated by multiple meta-analyses, while its impact on mental health, exercise capacity, and muscle adaptation in adults is increasingly attracting attention. However, heterogeneity persists across study findings, and the dose–response relationships as well as underlying mechanisms require further elucidation.

### 3.1. Fluoride Exposure and Neuropsychiatric Health

Multiple studies have investigated the effects of fluoride exposure on the central nervous system. A cross-sectional study involving 1169 adult participants assessed depression and anxiety using urinary fluoride levels and the PHQ-2 and GAD-2 scales, revealing that the prevalence of depression (3.17%) and anxiety (4.19%) showed no significant direct association with urinary fluoride; however, an interaction between urinary fluoride and income was observed, potentially influencing depression levels [34,35]. In contrast, among pediatric populations, a systematic review and meta-analysis encompassing 74 cross-sectional and prospective cohort studies identified a significant negative association between fluoride exposure and children’s IQ scores. This association dissipated when water fluoride concentrations were below 1.5 mg/L; however, urinary fluoride analysis continued to demonstrate a negative correlation even at lower concentrations, suggesting a potential risk of fluoride to neurodevelopment in children [33,36,37].

### 3.2. Fluoride Exposure and Sports Performance

The toxic effects of fluoride on the motor system extend to muscle function and exercise capacity. A systematic review indicated that fluoride impairs muscle contractility, endurance, and precision through disruption of calcium signaling; children in high-fluoride exposure regions exhibited an approximately 20% reduction in grip strength, prolonged reaction time, decreased maximal oxygen consumption (VO_2_ max), and presented with delayed motor milestones and neuromuscular deficits [38,39]. A randomized controlled study further quantified the impact of fluoride on muscle adaptation: healthy individuals undergoing resistance training were stratified by fluoride intake level, with the high-fluoride group demonstrating significantly lower gains in quadriceps cross-sectional area (+4.2% vs. +5.4%) and squat 1-RM (+12.5% vs. +15.2%) compared to the low-fluoride group, accompanied by attenuated osteocalcin response, elevated myostatin, and reduced electromyography gains. Changes in urinary fluoride were negatively correlated with muscle adaptation indicators (r = −0.42), suggesting that fluoride exposure dose-dependently impairs morphological and neural adaptation of skeletal muscle [40,41,42].

### 3.3. Effects of Fluoride on Metabolic and Respiratory Function During Exercise

Fluoride may also affect metabolic and respiratory health during exercise. A review focusing on endurance athletes indicated that fluoride enters the body through drinking water, fluoride-containing products, and sports beverages; during high-intensity training, athletes with high fluoride exposure were more susceptible to respiratory symptoms, including cough, shortness of breath, and wheezing, with symptom severity increasing alongside fluoride exposure levels and resulting in diminished athletic performance [43]. Furthermore, fluoride serves as a quintessential double-edged sword: appropriate intake (0.5–1.0 ppm in drinking water) confers anticariogenic benefits, whereas excessive intake leads to fluorosis [44,45]. Its effects on the motor system may be mediated through interference with energy metabolism, electrolyte balance, and oxidative stress pathways.

### 3.4. Fluoride Exposure, Skeletal Health, and Fluorosis Risk

Chronic excessive fluoride intake is directly attributable to endemic fluorosis, which manifests primarily as damage to the skeletal system. Although animal experiments have elucidated the disruptive effects of fluoride on bone metabolism signaling pathways, human studies have predominantly focused on epidemiological associations. Patients with skeletal fluorosis exhibit abnormal bone mineral density, joint deformities, and increased fracture risk, which are closely correlated with fluoride concentrations in drinking water [46,47,48]. Recent studies have further revealed that even in populations without skeletal fluorosis, fluoride exposure may influence bone metabolism markers and potentially modulate bone health through interplay with exercise.

### 3.5. Effects of Exercise on Fluoride Metabolism: Benefits and Potential Risks

Exercise alters fluoride absorption, distribution, and excretion in an intensity-dependent manner. In healthy adults, moderate-intensity exercise following fluoride ingestion increases peak plasma fluoride concentration (Cmax) and area under the curve (AUC), suggesting enhanced systemic absorption, while both moderate and vigorous exercise tend to reduce urinary fluoride excretion, possibly due to decreased renal blood flow and increased tubular reabsorption [49,50,51]. Athletes engaged in American football or mixed martial arts exhibit lower urinary fluoride levels than non-athletic controls, likely reflecting exercise-induced skeletal adaptations that promote fluoride deposition in bone [44,51,52].

Conversely, these same pharmacokinetic changes may pose risks under chronic high-fluoride exposure. Increased absorption and reduced excretion could theoretically aggravate fluoride retention. Indeed, high-intensity interval training in fluorosis-resistant mice led to greater femoral fluoride accumulation, indicating that exercise intensity and genetic background modulate fluoride deposition [31,53]. Moreover, athletes in fluoride-endemic regions may have an increased body fluoride burden due to exercise-induced skeletal uptake [44,52]. Thus, the net effect of exercise on fluorosis depends on a complex interplay of intensity, duration, baseline fluoride exposure, and individual susceptibility. Future research should clarify whether certain exercise regimens inadvertently worsen fluorosis, especially in vulnerable populations.

## 4. Animal Models and Exercise Protocols in Fluorosis Research

Animal models serve as a cornerstone for investigating the pathogenesis of fluorosis and the effects of exercise intervention. Existing studies have predominantly utilized rodents (mice and rats) exposed to varying doses of sodium fluoride (NaF) via drinking water or gavage to simulate chronic human fluoride exposure, combined with interventions such as forced treadmill running or voluntary wheel-running, to systematically evaluate the multi-organ protective effects of exercise against fluorosis [54]. Key modeling parameters include animal strain, fluoride exposure dose and duration, and exercise type and intensity; heterogeneity in these factors directly influences the comparability and extrapolative validity of study findings. Significant differences in susceptibility to fluoride exposure exist across animal strains, while the modality (forced vs. voluntary), intensity (moderate-intensity continuous exercise vs. high-intensity interval training), and synchronization of exercise interventions with fluoride exposure are critical considerations in model design. This section systematically reviews the methodologies for establishing fluorosis animal models, exercise intervention protocols, and associated modeling considerations, thereby providing a reference for future study design.

**Figure 2:**
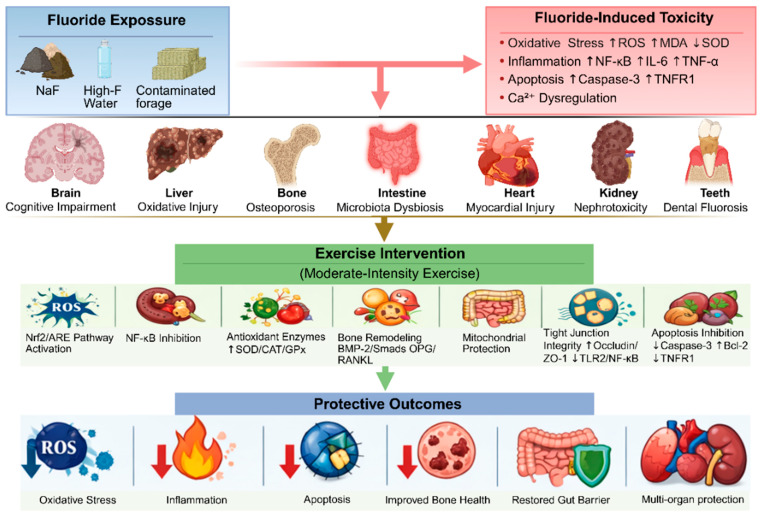
Mechanisms by which exercise mitigates fluoride-induced multi-organ toxicity. Fluoride exposure triggers oxidative stress (↑ROS/MDA, ↓SOD/CAT/GPx), inflammation (↑NF-κB/IL-6/TNF-α), apoptosis (↑Caspase-3/TNFR1), and Ca^2+^ dysregulation. Moderate-intensity exercise activates the Nrf2/ARE pathway, inhibits NF-κB signaling, enhances antioxidant enzymes, promotes bone remodeling via BMP-2/Smads and OPG/RANKL, protects mitochondrial function, maintains intestinal tight junction integrity (↑Occludin/ZO-1, ↓TLR2/NF-κB), and inhibits apoptosis (↓Caspase-3, ↑Bcl-2). These concerted mechanisms confer organ-specific protection against fluoride-induced damage in the brain, liver, bone, intestine, heart, kidneys, and teeth. Abbreviations are defined in the main text. The arrow symbols (↑ = upregulation/activation, ↓ = downregulation/inhibition).

Integrated molecular framework—Across all studied organs, three core pathways emerge as the primary mediators of exercise-induced protection against fluoride toxicity: (i) activation of the Nrf2/ARE antioxidant pathway, leading to upregulation of SOD, CAT, and GPx and reduction of ROS and MDA; (ii) suppression of NF-κB signaling, resulting in decreased pro-inflammatory cytokines (IL-6, TNF-α) and increased anti-inflammatory IL-10; and (iii) inhibition of apoptosis via downregulation of caspase-3 and TNFR1, and upregulation of Bcl-2. Tissue-specific pathways include BMP-2/Smads and OPG/RANKL/RANK in bone, and TLR2/NF-κB in intestine. These mechanisms are schematically integrated in Figure 2.

### 4.1. Establishment of Fluorosis Animal Models

#### 4.1.1. Commonly Used Animal Strains and Selection Considerations

Existing studies have predominantly utilized rodents, with mice being the most common, particularly the Institute of Cancer Research (ICR) mouse strain. Due to their sensitivity to fluoride exposure and well-defined genetic background, ICR mice have been extensively employed in fluorosis research [55,56,57,58]. Some studies have utilized rat models to investigate behavioral alterations associated with fluorosis and the interactive effects of exercise and heat stress [59]. Notably, significant differences in susceptibility to fluoride exposure exist among mouse strains: Fernandes et al. categorized mice into fluoride-sensitive (S) and fluoride-resistant (R) cohorts, revealing distinct responses in fluoride metabolism, oxidative stress, and exercise capacity. Fluoride-sensitive mice exhibited more pronounced oxidative stress and greater impairment in exercise capacity following fluoride exposure, underscoring genetic background as a critical consideration in model design [6,31].

#### 4.1.2. Fluoride Exposure Routes and Dosage

Fluoride exposure is primarily achieved through the drinking water route, mirroring the real-world scenario of human intake via contaminated water. The majority of studies have dissolved NaF in distilled water, with exposure concentrations typically ranging from 50 to 100 mg/L. For instance, Li et al. administered 100 mg/L NaF to mice in an 8-week study, successfully inducing the skeletal fluorosis phenotype [2]; similarly, Chai et al. employed 100 mg/L NaF in a 6-month study to establish a chronic fluorosis model, observing significant neurobehavioral alterations [60]. Fernandes et al. utilized 50 ppm NaF to investigate differences between fluoride-sensitive and fluoride-resistant strains, demonstrating that this dosage was sufficient to significantly elevate plasma and bone fluoride concentrations in both strains [6]. Some studies have employed gavage administration to achieve precise dose control; for example, 24 mg/kg NaF was administered to observe neuroinflammatory responses [28].

#### 4.1.3. Modeling Duration and Experimental Timeline

Modeling duration varies according to research objectives, ranging from 8 weeks to 12 months. Short-term models (8 weeks) are primarily employed to observe early alterations such as bone metabolism and oxidative stress; studies by Li et al. demonstrated that 8-week fluoride exposure was sufficient to induce histological alterations and signaling pathway abnormalities in bone [2,61]. Medium-term models (3–6 months) are widely utilized in systemic investigations of neurotoxicity, liver injury, and intestinal microbiota dysbiosis. For instance, Liu et al. observed fluoride-induced hepatocyte apoptosis at 3 and 6 months [30], while intestinal studies employed a 6-month modeling period to examine microbiota alterations [62]. Long-term models (12 months) are employed to investigate chronic cumulative effects such as dental fluorosis; research has shown that 12-month fluoride exposure significantly increases incisor fluoride content and induces ameloblast apoptosis in mice [63,64,65,66,67,68]. Modeling requires consideration of the synchronization between fluoride exposure and exercise intervention, with most studies adopting concurrent intervention designs to simulate real-world scenarios where exercise and fluoride exposure co-exist.

### 4.2. Exercise Intervention Modalities

#### 4.2.1. Exercise Types and Apparatus

Current research has predominantly employed two exercise intervention modalities: forced treadmill running and voluntary wheel-running. Treadmill running, which allows precise control over exercise intensity, duration, and frequency, constitutes the mainstream choice for mechanistic studies. Multiple studies have adopted a protocol consisting of a 5 min cool-down at 5 m/min, 20 min exercise at 10–12 m/min, and 5 min cool-down at 5 m/min, performed 5 days per week. This regimen has been corroborated to exert multi-organ protective effects in fluorosis mice [30,65]. Some studies have employed high-intensity interval training (HIIT) to investigate the effects of different exercise intensities, with protocols involving high-intensity interval treadmill running over an 8-week period [31]. Voluntary wheel-running more closely mimics the natural locomotor behavior of animals and minimizes the potential stress interference associated with forced exercise; however, it is characterized by substantial inter-individual variability in exercise volume. A study on renal oxidative stress employed voluntary wheel-running with subgroups stratified by daytime, nighttime, and all-day exercise, revealing that the extent of renal injury amelioration was positively correlated with running distance [69]. Swimming training has also been applied in earlier studies; for instance, Basha et al. employed swimming training combined with heat stress to observe cognitive function alterations in fluoride-exposed rats [60].

#### 4.2.2. Exercise Intensity and Frequency

The determination of exercise intensity should take into account the physiological characteristics of the animal model and the specific experimental objectives. Moderate-intensity continuous exercise (e.g., 10–12 m/min) has been demonstrated to exert multi-organ protective effects in fluorosis mice, including neuroprotection, hepatoprotection, and bone protection [2,29]. High-intensity interval training has been shown to significantly increase bone fluoride accumulation in fluoride-resistant mice, suggesting that exercise intensity may influence the systemic distribution of fluoride, with high-intensity exercise potentially promoting fluoride deposition in bone tissue [31]. Exercise frequency is typically set at 5 days per week, with daily sessions lasting 20–30 min, and intervention durations ranging from 8 weeks to 12 months. Some studies have adopted daily exercise protocols; for instance, voluntary wheel-running allows animals free access to activity throughout the day, enabling the recording of daily running distance and subsequent analysis of dose–response relationships with protective effects [70].

#### 4.2.3. Synchronization of Exercise and Fluoride Exposure

The majority of studies have employed protocols with concurrent fluoride exposure and exercise intervention to evaluate the preventive or alleviative effects of exercise on fluorosis. For instance, Chai et al. conducted treadmill exercise during a 6-month fluoride exposure period to investigate the neuroprotective effects of exercise [29]. Similarly, intestinal studies utilized a 6-month synchronized intervention design, demonstrating that exercise alleviates fluoride-induced intestinal damage and microbiota dysbiosis [65]. Although some studies have adopted designs with fluoride exposure followed by exercise intervention, such protocols remain relatively scarce in the literature. Modeling must also account for animal age and sex factors: most studies have selected 3-week-old juvenile mice to simulate fluoride exposure during the growth and development period [30,71], while some investigations have included both sexes to explore sex differences. For example, a renal study employed an equal number of male and female mice but did not report sex-specific outcomes [72].

### 4.3. Modeling Considerations and Limitations

Significant heterogeneity exists among current animal models in terms of fluoride exposure dose, duration, and exercise modalities, warranting caution when directly comparing study findings [73]. While forced exercise allows precise control over exercise parameters, it may introduce stress-related confounding that influences fluoride metabolism and oxidative stress markers. It has been shown that forced exercise itself can elevate stress hormone levels, potentially interacting with fluoride exposure. In contrast, voluntary wheel-running better aligns with animals’ natural behavior and minimizes stress interference; however, individual variations in exercise volume are substantial and require normalization through recorded running distance. Renal studies have demonstrated that the degree of improvement in oxidative stress markers correlates significantly with exercise distance [69,74]. Genetic background differences across strains may influence susceptibility to fluoride and responsiveness to exercise, as evidenced by marked disparities in fluoride metabolism, oxidative stress, and exercise capacity between fluoride-sensitive and fluoride-resistant strains [31,75], This underscores the need for future studies to prioritize strain selection and the regulatory role of genetic factors. Additionally, modeling should integrate considerations such as animal age, sex, and the synchronization of fluoride exposure with exercise intervention to enhance model reliability and extrapolative validity. Currently, systematic comparisons between different exercise modalities (forced vs. voluntary) and intensities (moderate vs. high) within the same model remain lacking [76], representing an important direction for future research. A summary of experimental models and exercise protocols employed in fluorosis research is presented in Table 1.

As summarized in Table 1, animal studies consistently demonstrate that regular moderate-intensity exercise confers protective effects across multiple organ systems—including the nervous system, liver, bone, intestine, heart, kidneys, and teeth—by reducing oxidative stress, attenuating inflammatory responses, inhibiting apoptosis, and modulating key signaling pathways (e.g., Nrf2/ARE, BMP-2/Smads, and OPG/RANKL/RANK). As shown in Figure 2, these findings provide mechanistic insights into the therapeutic potential of exercise against fluoride-induced multi-organ damage.

## 5. Insights from Animal Studies: Organ-Specific Protective Effects of Exercise

**Figure 3:**
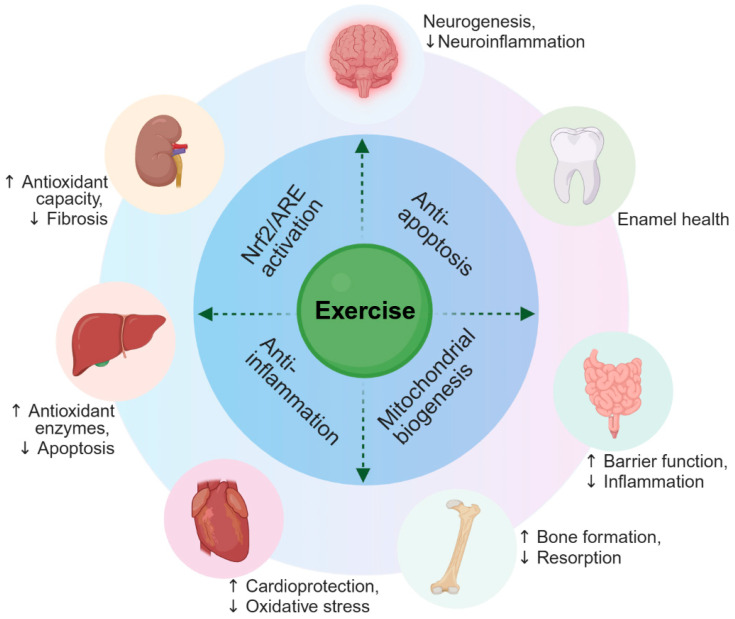
Schematic overview of exercise-induced multi-organ benefits. Regular moderate-intensity exercise promotes health across multiple organ systems through activation of antioxidant pathways (Nrf2/ARE), enhancement of mitochondrial function, suppression of inflammation, and inhibition of apoptosis. Key beneficial effects are indicated for each organ. Arrows indicate increase (↑) or decrease (↓) relative to controls.

Animal experiments have provided critical evidence for understanding the ameliorative effects and mechanisms of exercise against fluorosis. Existing studies have primarily utilized rodent models exposed to varying doses of sodium fluoride (NaF) via drinking water or gavage, combined with interventions such as forced treadmill running or voluntary wheel-running, to systematically evaluate the toxic effects of fluoride exposure on multi-organ systems and the regulatory effects of exercise. Research encompasses target organs, including the nervous system, liver, bone, intestine, heart, kidneys, and teeth, and involves multiple dimensions such as oxidative stress, inflammatory responses, apoptosis, signaling pathway regulation, and intestinal microbiota [54,55]. Collectively, regular moderate-intensity exercise demonstrates multi-organ protective effects in various fluorosis animal models, with mechanisms primarily associated with enhanced antioxidant defense, inhibition of inflammation, regulation of bone metabolism signaling pathways, and maintenance of intestinal barrier function (Figure 3). Notably, differences in susceptibility to fluoride exposure exist among mouse strains, and the effects of exercise on fluoride metabolism may be influenced by genetic background [76].

### 5.1. Neuroprotective Effects

Fluoride exposure impairs learning and memory capacity and induces structural and functional damage to the central nervous system. A 6-month study revealed that mice administered 100 mg/L NaF in drinking water exhibited deficits in learning and memory, accompanied by ultrastructural damage to mitochondria and the myelin sheath in brain tissue, as well as a reduced neuronal count [56,77]. Mechanistically, fluoride exposure significantly increased the number of M1-type microglia in the hippocampus, indicating neuroinflammation, and altered the neuroactive ligand–receptor interaction pathway [28]. Furthermore, fluoride disrupted miRNA expression, particularly miR-206-3p, and altered pregnenolone (PREG) metabolism, contributing to neuronal apoptosis and cognitive decline [29,58,59].

Exercise is known to enhance cognitive function and promote neuroplasticity through stimulation of neurogenesis, upregulation of neurotrophic factors, and suppression of neuroinflammation [21,24,25,27]. In fluoride-exposed mice, treadmill exercise significantly ameliorated cognitive deficits and structural impairments [29,60]. Metabolomic analysis suggested that PREG may serve as a biomarker for exercise-mediated alleviation of fluoride neurotoxicity, while miRNA sequencing and in vitro experiments confirmed that miR-206-3p attenuates cell death through regulation of JunD and HDAC4 [29,58]. Additionally, exercise reversed the increase in M1-type microglia and modulated the neuroactive ligand–receptor interaction pathway [28]. These findings demonstrate that exercise counteracts fluoride neurotoxicity by targeting multiple molecular pathways, including miRNA regulation, neuroinflammation suppression, and metabolic modulation.

### 5.2. Hepatoprotective Effects

Fluoride-induced hepatotoxicity involves oxidative damage and apoptosis. A study spanning 3 to 6 months demonstrated that exposure to 100 mg/L NaF resulted in increased hepatocyte pyknosis and apoptosis in mice, as evidenced by elevated TUNEL-positive cells [30,61]. Molecular studies revealed that fluoride induces hepatocyte apoptosis through the TNFR1 signaling pathway, accompanied by decreased glutathione peroxidase (GPx) activity and reduced total antioxidant capacity (T-AOC) [30,61,62].

Regular moderate-intensity exercise is well-documented to enhance hepatic antioxidant defense through upregulation of antioxidant enzymes and activation of Nrf2/ARE signaling, while also suppressing NF-κB-mediated inflammation [19,20,25,26,27]. In fluoride-exposed mice, treadmill exercise reversed hepatocyte apoptosis and restored the aberrant expression of TNFR1 pathway-related molecules [30,61]. Additional research found that exercise alleviated hepatic oxidative stress, manifested by restoration of GPx activity and T-AOC, as well as prevention of fluoride-induced abnormal elevations in superoxide dismutase (SOD) and catalase (CAT) activities [62]. These results indicate that exercise protects the liver from fluoride toxicity by enhancing antioxidant capacity and inhibiting the TNFR1 apoptotic pathway.

### 5.3. Skeletal Protective Effects

The toxic effects of fluoride on bone are primarily manifested as bone metabolic disorders and structural abnormalities. An 8-week study found that exposure to 100 mg/L NaF increased bone fluoride accumulation in mice, altered bone histological structure, elevated alkaline phosphatase (ALP) and tartrate-resistant acid phosphatase (TRACP) activities, and induced oxidative stress in bone tissue (characterized by increased ROS and MDA levels and decreased antioxidant enzyme activities) [2,63]. qRT-PCR and Western blot analyses revealed that fluoride upregulated the expression of genes and proteins associated with the BMP-2/Smads and OPG/RANKL/RANK signaling pathways, indicating disrupted bone remodeling [2,64].

Mechanical loading from exercise is known to enhance bone remodeling through activation of Wnt/β-catenin and BMP-Smad signaling pathways, promoting osteoblast activity and improving bone mineral density [16,17,18,22]. In fluorosis mice, moderate-intensity treadmill exercise significantly alleviated fluoride-induced alterations in bone histology, reduced bone fluoride content, and normalized ALP and TRACP activities [2,64]. Exercise also restored the expression of genes and proteins in the BMP-2/Smads and OPG/RANKL/RANK pathways, correcting the imbalance caused by fluoride [2]. Interestingly, a study in fluoride-sensitive and fluoride-resistant mouse strains showed that high-intensity interval training (HIIT) significantly increased femoral fluoride accumulation in fluoride-resistant mice, suggesting that exercise intensity and genetic background may influence fluoride deposition in bone tissue [31].

### 5.4. Intestinal Protective Effects

Fluorosis is frequently accompanied by gastrointestinal symptoms, with underlying mechanisms involving impairment of intestinal morphology and function, as well as microbiota dysbiosis. A 6-month study revealed that exposure to 100 mg/L NaF in mice resulted in decreased duodenal villus height (VH) and VH/crypt depth ratio, reduced mRNA expression of tight junction proteins (Occludin, ZO-1, Claudin-1), and elevated mRNA expression of pro-inflammatory cytokines (IL-1β, IL-6, TNF-α, TLR2, NF-κB p65) [65]. 16S rDNA sequencing demonstrated that fluoride exposure altered intestinal microbiota composition, decreasing the abundances of Campylobacterota and Firmicutes while increasing Bacteroidetes abundance, and affected the abundance of 13 bacterial genera [65,66].

Regular moderate exercise is recognized as maintaining intestinal mucosal function and immune homeostasis by enhancing barrier integrity, reducing inflammation, and promoting a healthy gut microbiota composition through suppression of TLR2/NF-κB signaling [25,26,65]. In fluoride-exposed mice, treadmill exercise effectively restored intestinal morphological structure and barrier function, inhibited inflammatory responses, and reversed microbial dysbiosis [65,66]. Exercise restored the mRNA levels of tight junction proteins (Occludin, ZO-1, Claudin-1) in the duodenum and colon, normalized the expression of pro-inflammatory cytokines and TLR2/NF-κB p65, and rebalanced the gut microbial ecosystem by restoring the abundances of Epsilonbacteraeota and Firmicutes while reducing Bacteroidetes [65].

### 5.5. Cardioprotective Effects

Fluoride exposure induces cardiac inflammatory responses and structural damage. A 6-month study demonstrated that exposure to 100 mg/L NaF caused myocardial morphological injury in mice, with transmission electron microscopy revealing ultrastructural abnormalities and immunofluorescence staining indicating increased expression of the macrophage marker CD68 [67,68]. qRT-PCR analysis showed upregulated mRNA expression of pro-inflammatory cytokines (IL-6, IL-1β, and TNF-α) and downregulated expression of the anti-inflammatory cytokine IL-10 [67,68].

Moderate-intensity exercise is known to promote cardiac function and immune homeostasis by enhancing myocardial antioxidant capacity, reducing baseline inflammation, and regulating macrophage polarization [15,22,25,26,27]. In fluoride-exposed mice, treadmill exercise significantly attenuated myocardial injury, decreased CD68 expression and pro-inflammatory cytokine levels, and restored IL-10 expression [67,68]. These findings demonstrate that exercise mitigates fluoride-induced cardiac inflammation and structural damage through suppression of NF-κB-mediated inflammation and regulation of macrophage polarization.

### 5.6. Renoprotective Effects

Fluoride-induced renal injury is closely associated with oxidative stress. A 6-month study utilizing a voluntary wheel-running exercise model revealed that exposure to 100 mg/L NaF caused structural damage to kidney tissue in mice, elevated serum blood urea nitrogen (BUN) and uric acid (UA) levels, and increased levels of ROS, MDA, H_2_O_2_, and CAT activity in renal tissue, while decreasing GSH content and T-SOD and GSH-PX activities [69,70,71].

Regular exercise is well-established to improve renal function and reduce oxidative stress through enhancement of antioxidant enzyme activities, increased glutathione levels, and promotion of mitochondrial health [25,70,71]. In fluoride-exposed mice, voluntary wheel-running exercise effectively reversed fluoride-induced structural and biochemical alterations in the kidney, with the degree of renal injury amelioration positively correlated with running distance [69]. Exercise restored GSH content, T-SOD, and GSH-PX activities, and reduced ROS, MDA, and H_2_O_2_ levels, demonstrating a dose-dependent protective effect against fluoride nephrotoxicity [69].

### 5.7. Dental Protective Effects

Dental fluorosis represents an early manifestation of fluorosis, with its pathogenesis involving oxidative stress and apoptosis in ameloblasts. A 12-month study revealed that exposure to 100 mg/L NaF significantly increased incisor fluoride content in mice, elevated urinary CTX-I levels, and increased ROS and MDA levels in incisor tissue, while decreasing the activities of CAT, GSH-Px, T-SOD, and T-AOC [72,73,74]. Ameloblast apoptosis was increased, accompanied by upregulated mRNA expression of p38MAPK, JNK, IKK-β, and Caspase3, and downregulated mRNA expression of Bcl-2 and SOD1 [74].

Regular physical activity is known to reduce systemic oxidative stress and inflammation, which may indirectly benefit oral tissues [20,25]. In fluoride-exposed mice, forced treadmill exercise significantly suppressed oxidative stress and apoptotic alterations in ameloblasts [72,73,74]. Exercise reduced ROS and MDA levels, restored antioxidant enzyme activities, and normalized the expression of apoptosis-related genes (↓p38MAPK, ↓JNK, ↓IKK-β, ↓Caspase3; ↑Bcl-2, ↑SOD1) [78]. These findings suggest that regular exercise may serve as a convenient therapy for dental fluorosis by mitigating oxidative damage and apoptosis in ameloblasts.

As summarized in Table 2, animal studies consistently demonstrate that regular moderate-intensity exercise confers protective effects across multiple organ systems—including the nervous system, liver, bone, intestine, heart, kidneys, and teeth—by reducing oxidative stress, attenuating inflammatory responses, inhibiting apoptosis, and modulating key signaling pathways (e.g., Nrf2/ARE, BMP-2/Smads, and OPG/RANKL/RANK). As shown in Figure 2, these findings provide mechanistic insights into the therapeutic potential of exercise against fluoride-induced multi-organ damage.

## 6. Conclusions

**Figure 4:**
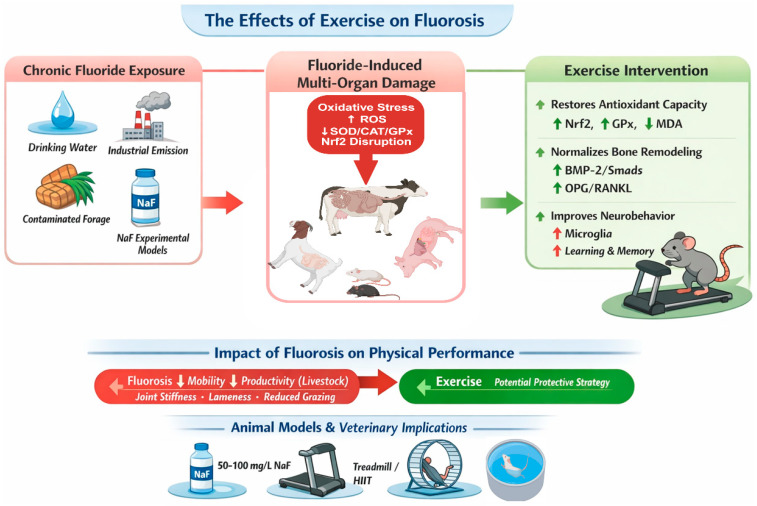
Schematic overview of the effects of exercise on fluorosis. The diagram summarizes fluoride sources, fluoride-induced multi-organ damage (oxidative stress), exercise interventions (antioxidant enhancement, bone remodeling, neurobehavioral improvement), the impact of fluorosis on physical performance, and veterinary implications. Abbreviations are defined in the main text.

This systematic review synthesizes current evidence that regular moderate-intensity exercise can mitigate multi-organ damage induced by fluorosis in animal models. Figure 4 provides a schematic overview of the bidirectional relationship between exercise and fluorosis, summarizing fluoride sources, multi-organ damage, exercise interventions, and veterinary implications. The primary protective mechanisms converge on activation of the Nrf2/ARE antioxidant pathway, suppression of NF-κB-mediated inflammation, inhibition of apoptosis, regulation of bone remodeling (BMP-2/Smads, OPG/RANKL/RANK), and maintenance of intestinal barrier integrity (TLR2/NF-κB). Human studies, although limited, indicate that exercise modulates fluoride pharmacokinetics in an intensity-dependent manner, with both beneficial outcomes (enhanced bone fluoride deposition) and potentially adverse consequences (increased absorption and retention).

However, the current body of evidence is largely derived from rodent studies using fluoride doses (50–100 mg/L NaF) that far exceed typical environmental exposure levels in humans and livestock, and this high-dose paradigm limits direct translational applicability. Considerable heterogeneity also exists among models regarding exposure duration, exercise protocols, and outcome measures, which precludes meta-analysis. Moreover, human data remain scarce and cross-sectional, lacking long-term intervention trials.

We propose that exercise should be viewed as a bidirectional modulator of fluoride toxicity. While moderate-intensity exercise confers clear multi-organ protection in controlled animal settings, its net effect in real-world fluoride-exposed populations may depend on exercise intensity, genetic background, and baseline fluoride burden. Future research should adopt more environmentally relevant fluoride exposure regimens (e.g., lower doses, longer durations) in animal models, conduct well-designed human cohort studies to assess the risk–benefit balance of exercise in fluorosis-endemic regions, and explore personalized exercise prescriptions based on individual fluoride exposure levels. Ultimately, exercise could serve as a low-cost, adjunctive strategy to improve livestock health in fluoride-contaminated areas, but caution is warranted to avoid inadvertent exacerbation of fluoride retention.

## Figures and Tables

**Table 1:** Summary of experimental models and exercise protocols in fluorosis research.

Animal Strain	Fluoride Exposure Route	Fluoride Dose/Concentration	Exposure Duration	Exercise Type	Exercise Protocol	Main Outcome Measures	References
Mouse	Drinking water (NaF)	100 mg/L	6 months	Treadmill exercise	Concurrent with exposure; 5 min warm-up + 20 min running + 5 min cool-down, 5 days/week	Learning and memory; Neuronal structure; miRNA; Metabolites	[29]
Fluorosis-susceptible	Drinking water (NaF)	50 ppm	8 weeks	HIIT treadmill	8 weeks; High-intensity interval training	Bone/liver fluoride content; Blood glucose; Oxidative stress	[31]
ICR mouse	Drinking water (NaF)	100 mg/L	3–6 months	Treadmill exercise	Concurrent with exposure; 5 days/week	Hepatocyte apoptosis; TNFR1 pathway	[30]
ICR mouse	Gavage (NaF)	24 mg/kg	8 weeks	Treadmill exercise	8 weeks; 5 days/week	Microglial activation; Hippocampal transcriptome	[28]
ICR mouse	Drinking water (NaF)	100 mg/L	8 weeks	Treadmill exercise	8 weeks; 5 days/week	Bone histology; Bone fluoride content; ALP/TRACP; BMP-2/Smads pathway; OPG/RANKL/RANK pathway	[2]
Rat	Drinking water (NaF)	600 ppm	2 weeks	swimming training	2 weeks; Daily swimming	Cognitive function; Brain/bone/plasma fluoride content; Oxidative stress	[60]

Fluoride concentrations are presented as reported in the original studies (1 ppm ≈ 1 mg/L for aqueous solutions). Abbreviations: HIIT, high-intensity interval training; ICR, Institute of Cancer Research; NaF, sodium fluoride; ALP, alkaline phosphatase; BMP-2, bone morphogenetic protein-2; OPG, osteoprotegerin; RANK, receptor activator of nuclear factor-κB; RANKL, receptor activator of nuclear factor-κB ligand; TNFR1, tumor necrosis factor receptor 1; TRACP, tartrate-resistant acid phosphatase. Concurrent exposure indicates that fluoride and exercise interventions were administered simultaneously throughout the experimental period.

**Table 2:** Multi-organ protective effects of exercise against fluoride toxicity in animal models.

Organ System	Fluoride-Induced Damage Manifestations	Ameliorative Effects of Exercise Intervention	Involved Molecular Mechanisms/Pathways	Reference
Nervous System	Learning and memory deficits; Increased M1-type microglia in hippocampus; Ultrastructural damage to mitochondria and myelin sheath; Reduced neuronal count	Cognitive function recovery; Decreased M1-type microglia; Restoration of mitochondrial and myelin sheath structure	Regulation of miR-206-3p; Alterations in pregnenolone (PREG) metabolism; Neuroactive ligand–receptor interaction pathway	[28,29,60]
Liver	Hepatocyte apoptosis (nuclear condensation, TUNEL-positive); Oxidative damage (↓GPx activity, ↓T-AOC)	Reversal of apoptosis; Restoration of GPx and T-AOC; Normalization of SOD/CAT activities	Inhibition of TNFR1 apoptotic pathway; Enhancement of antioxidant system	[30,62]
Bone	Abnormal bone histology (osteosclerosis/osteoporosis); ↑Fluoride accumulation in bone; ↑ALP/TRACP activities; Oxidative stress (↑MDA)	Improved bone histology; ↓Fluoride content in bone; Normalization of ALP/TRACP activities; ↓MDA levels	Restoration of BMP-2/Smads signaling; Balancing of OPG/RANKL/RANK pathway	[2]
Intestine	Decreased villus height (VH) and VH/crypt depth ratio; ↓Tight junction protein expression (Occludin, ZO-1, Claudin-1); ↑Pro-inflammatory cytokines (IL-1β, IL-6, TNF-α); Microbiota dysbiosis	Restored intestinal morphology and barrier function; ↓Inflammatory responses; Reversed microbiota dysbiosis	Inhibition of TLR2/NF-κB pathway; Restoration of tight junction proteins; Modulation of gut microbiota composition	[65,66]
Heart	Myocardial morphological injury; Ultrastructural abnormalities; ↑Macrophage marker CD68; ↑Pro-inflammatory cytokines (IL-6, IL-1β, TNF-α); ↓Anti-inflammatory cytokine IL-10	Attenuated myocardial injury; ↓CD68 expression; ↓Pro-inflammatory cytokine levels; Restored IL-10 expression	Suppression of NF-κB-mediated inflammation; Regulation of macrophage polarization	[67,68]
Kidney	Structural damage to kidney tissue; ↑Serum BUN and UA; ↑ROS, MDA, H_2_O_2_, and CAT activity in renal tissue; ↓GSH content, T-SOD, and GSH-PX activities	Reversed structural and biochemical alterations; Amelioration correlated with exercise distance	Activation of Nrf2/ARE pathway; Enhancement of antioxidant enzyme activities	[67,68,69]
Teeth	↑Incisor fluoride content; ↑Urinary CTX-I; ↑ROS and MDA in incisor tissue; ↓CAT, GSH-Px, T-SOD, T-AOC activities; Ameloblast apoptosis	Suppressed oxidative stress and apoptosis	Inhibition of p38MAPK/JNK/IKK-β/Caspase3 pathway; Upregulation of Bcl-2 and SOD1	[72,73,74]
Shared Mechanisms	Systemic oxidative stress; Inflammation; Apoptosis	Enhanced antioxidant capacity; Reduced inflammation; Inhibited apoptosis	Nrf2/ARE pathway activation; NF-κB suppression; Mitochondrial protection	[2,62]

Data are compiled from studies examining the effects of exercise against fluoride-induced multi-organ damage in animal models. Arrows indicate increase (↑) or decrease (↓) relative to controls. Abbreviations: ALP, alkaline phosphatase; ARE, antioxidant response element; BUN, blood urea nitrogen; CAT, catalase; CD, crypt depth; GPx, glutathione peroxidase; GSH, glutathione; GSH-PX, glutathione peroxidase; IKK-β, IκB kinase beta; IL, interleukin; JNK, c-Jun N-terminal kinase; MDA, malondialdehyde; miRNA, microRNA; NF-κB, nuclear factor kappa-B; Nrf2, nuclear factor erythroid 2-related factor 2; OPG, osteoprotegerin; p38MAPK, p38 mitogen-activated protein kinase; PREG, pregnenolone; RANK, receptor activator of nuclear factor-κB; RANKL, receptor activator of nuclear factor-κB ligand; ROS, reactive oxygen species; SOD, superoxide dismutase; T-AOC, total antioxidant capacity; TLR2, Toll-like receptor 2; TNFR1, tumor necrosis factor receptor 1; TNF-α, tumor necrosis factor-alpha; TRACP, tartrate-resistant acid phosphatase; T-SOD, total superoxide dismutase; TUNEL, terminal deoxynucleotidyl transferase dUTP nick end labeling; UA, uric acid; VH, villus height; ZO-1, zonula occludens-1.

## Data Availability

No new data were created or analyzed in this study. Data sharing is not applicable to this article.

## Abbreviations

The following abbreviations are used in this manuscript:

ALP	Alkaline phosphatase
ARE	Antioxidant response element
ATP	Adenosine triphosphate
AUC	Area under the concentration–time curve
BMD	Bone mineral density
BMP-2	Bone morphogenetic protein-2
BUN	Blood urea nitrogen
CAT	Catalase
CD	Crypt depth
Cmax	Peak concentration
CTX-I	C-terminal telopeptide of type I collagen
GAD-2	Generalized Anxiety Disorder-2
GPx	Glutathione peroxidase
GSH	Glutathione
HE	Hematoxylin-eosin
HIIT	High-intensity interval training
ICR	Institute of Cancer Research
IL	Interleukin
JunD	Jun D proto-oncogene
HDAC4	Histone deacetylase 4
MDA	Malondialdehyde
MMA	Mixed martial arts
mRNA	Messenger RNA
miRNA	MicroRNA
NaF	Sodium fluoride
NF-κB	Nuclear factor kappa-B
Nrf2	Nuclear factor erythroid 2-related factor 2
OPG	Osteoprotegerin
PHQ-2	Patient Health Questionnaire-2
p38MAPK	p38 mitogen-activated protein kinase
PREG	Pregnenolone
qRT-PCR	Quantitative real-time polymerase chain reaction
RANK	Receptor activator of nuclear factor-κB
RANKL	Receptor activator of nuclear factor-κB ligand
ROS	Reactive oxygen species
SOD	Superoxide dismutase
T-AOC	Total antioxidant capacity
TEM	Transmission electron microscopy
TLR	Toll-like receptor
TNFR1	Tumor necrosis factor receptor 1
TNF-α	Tumor necrosis factor-alpha
TRACP	Tartrate-resistant acid phosphatase
TUNEL	Terminal deoxynucleotidyl transferase dUTP nick end labeling
UA	Uric acid
UF	Urinary fluoride
VH	Villus height
VO_2_ max	Maximal oxygen consumption
ZO-1	Zonula occludens-1
